# Pole balancing on the fingertip: model-motivated machine learning forecasting of falls

**DOI:** 10.3389/fphys.2024.1334396

**Published:** 2024-04-04

**Authors:** Minakshi Debnath, Joshua Chang, Keshav Bhandari, Dalma J. Nagy, Tamas Insperger, John G. Milton, Anne H. H. Ngu

**Affiliations:** ^1^ Department of Computer Science, Texas State University, San Marcos, TX, United States; ^2^ Department of Neurology, Dell Medical School, The University of Texas at Austin, Austin, TX, United States; ^3^ Oden Institute for Computational Engineering and Sciences, The University of Texas at Austin, Austin, TX, United States; ^4^ Department of Applied Mechanics, Faculty of Mechanical Engineering, Budapest University of Technology and Economics, Budapest, Hungary; ^5^ HUN-REN–BME Dynamics of Machines Research Group, Budapest, Hungary

**Keywords:** forecast, transfer learning, long short-term memory, pole balancing, synthetic data, micro-chaotic systems, physics-inspired physiological model, adverse events

## Abstract

**Introduction:** There is increasing interest in developing mathematical and computational models to forecast adverse events in physiological systems. Examples include falls, the onset of fatal cardiac arrhythmias, and adverse surgical outcomes. However, the dynamics of physiological systems are known to be exceedingly complex and perhaps even chaotic. Since no model can be perfect, it becomes important to understand how forecasting can be improved, especially when training data is limited. An adverse event that can be readily studied in the laboratory is the occurrence of stick falls when humans attempt to balance a stick on their fingertips. Over the last 20 years, this task has been extensively investigated experimentally, and presently detailed mathematical models are available.

**Methods:** Here we use a long short-term memory (LTSM) deep learning network to forecast stick falls. We train this model to forecast stick falls in three ways: 1) using only data generated by the mathematical model (synthetic data), 2) using only stick balancing recordings of stick falls measured using high-speed motion capture measurements (human data), and 3) using transfer learning which combines a model trained using synthetic data plus a small amount of human balancing data.

**Results:** We observe that the LTSM model is much more successful in forecasting a fall using synthetic data than it is in forecasting falls for models trained with limited available human data. However, with transfer learning, i.e., the LTSM model pre-trained with synthetic data and re-trained with a small amount of real human balancing data, the ability to forecast impending falls in human data is vastly improved. Indeed, it becomes possible to correctly forecast 60%–70% of real human stick falls up to 2.35 s in advance.

**Conclusion:** These observations support the use of model-generated data and transfer learning techniques to improve the ability of computational models to forecast adverse physiological events.

## 1 Introduction

There is increasing interest in leveraging physiological measurements to forecast adverse clinical events ranging from susceptibility to cardiac arrhythmias (Walker et al., 2017; Varshneya et al., 2021) to adverse surgical outcomes (Chen et al., 2021), predicting limb movements using electromyography (EMG) signals (Smirnov et al., 2021), understanding the effects of ankle weakness on human gait (Ong et al., 2019) and adverse events in human balancing tasks (Zgonnikova et al., 2016; Wang et al., 2022; Márquez et al., 2023). Studies over the last few years have utilized advances in machine learning and, more specifically, deep learning, to learn dynamical behaviors (Gilpin et al., 2020). However, in many physiological processes, the dynamics are exceedingly complex and may not be amenable to these techniques, especially when given a limited amount of data.

A case in point concerns the forecasting of falls for stick balancing on the fingertip (Márquez et al., 2023). The dynamics of the movements of the balanced stick are exceedingly complex and include on-off intermittency (Cabrera and Milton, 2002), Levy flights (Cabrera and Milton, 2004a), and micro-chaotic fluctuations (Milton et al., 2016; 2018). These dynamical signatures form the cornerstone upon which current theories of human balance and falls can be based (Insperger and Milton, 2021). Thus, it can be anticipated that strategies that forecast the fall of an inverted pendulum will translate into effective strategies for forecasting human falls. An important benchmark is how far in advance is it possible to forecast a fall? Clearly, the longer a fall can be forecasted ahead, the more time is available to activate strategies to mitigate the adverse effects of a fall (Nemeth et al., 2022).

Recently it was shown that by using traditional machine learning techniques (e.g., artificial neural networks, random forest, support vector machine, k-nearest neighbor), it is possible to forecast falls for human pole balancing on the fingertip by ≈0.83 s (Márquez et al., 2023) based on data from two pole balancers. However, it is known that machine learning model trained with small amount of data cannot generalize to the different skill levels of pole balancers, as reflected by different mean balance times. Pole balancing times can range from a few seconds to many minutes depending on the skill of the human balancer. Thus, fatigue limits the number of balance trials to <100 per day with a significant bias towards the less skilled balancers.

An attractive solution to this data scarcity problem is to use a mathematical model to generate any desired amount of synthetic stick falls and use this data to train a machine learning model to approximate the complex dynamics of stick falling. This strategy is similar in spirit to the use of physics-inspired models to investigate the effects of ankle plantar flexor weakness on gait (Ong et al., 2019), prediction of arrhythmias susceptibility (Varshneya et al., 2021), and to estimate limb movements using EMG signals (Smirnov et al., 2021). In all of these cases, synthetic data is generated via the relevant mathematical models in order to gain insights into the underlying physiology of the clinically-relevant system. These relevant mathematical models serve as a “digital twin” of the various systems in question (Kapteyn et al., 2020).

We approach the difficult problem of obtaining a generalized model for forecasting of pole falls limited by a small amount of real data by first generating synthetic pole movement data using a physiologically motivated mathematical model for pole balancing on the fingertip. This mathematical model for pole balancing has been developed and refined in our laboratory over the last 20 years (Cabrera and Milton, 2004b; Insperger and Milton, 2014; Milton et al., 2016; Milton et al., 2018; Milton and Insperger, 2019; Insperger and Milton, 2021; Nagy et al., 2023). Although not yet perfect, this model nonetheless reproduces the same balance time distribution and many of the frequency and kinematics-dependent properties as measured experimentally for human pole balancing. Next, we trained a stacked long short-term memory (LSTM) deep learning network on synthetic data generated using this model to learn the patterns leading to pole falling. Finally, we use transfer learning to fine-tune this model with a limited amount of real-world human pole-balancing data.

LSTMs are part of a class of recurrent neural network (RNN) deep-learning algorithms that have been used in time-series forecasting. Unlike traditional feed-forward neural networks, LSTMs are able to use their internal memory to process arbitrary sequences of inputs, capturing the temporal dynamics of the data. Moreover, deep learning models do not require hand-crafted feature engineering. The model can learn the latent representation inherent in the data via lots of examples/data. Designing good hand-crafted features requires expert knowledge and is a labor-intensive process. This particular deep neural network architecture has been used previously to train a model to recognize motion-related activities from a wristwatch over a period of time so that they can be better distinguished from others (Mauldin et al., 2018). A bi-directional LSTM has also been used in Çiçek et al. (2022) for the forecasting of human physical activity from smartphone sensors.

We conducted an extensive set of experiments to train a range of forecasting models with forecast times ranging from 0.85 to 2.35 s to assess the limit of forecasting in an unstable dynamic system using a combination of measured human stick balancing data, simulated data generated by a mathematical model for stick balancing and deep transfer learning for refinement. We show that it is possible to correctly forecast 60%–70% of real human falls up to 2.35 s in advance.

Our paper is organized as follows. First, we describe how real-world human pole balancing data is collected. Next, we present the mathematical model for human pole balancing and describe how the important parameters (time delay, sensory dead zone, and four reflex gains) were estimated. We describe how the mathematical model-generated data is pre-processed as suitable input to LSTM algorithm, and how the unbalanced nature of the fall events (i.e., falls being “rare” events relative to non-falls) was overcome. In the next section, we show how transfer learning was utilized to combine the benefits of synthetic data generation with real-world examples for improved accuracy in event anticipation. Finally, we discuss the implications of our findings and future directions for this approach for fall forecasting.

## 2 Materials and methods

### 2.1 Data collection

**FIGURE 1:**
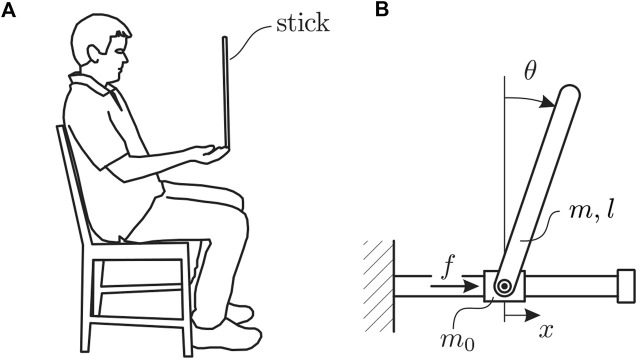
Human pole balancing illustrating both **(A)** human position during task, and **(B)** the mathematical representation of the pole balancing behavior as an inverted pendulum-cart mechanical model.

Human pole balancing data collection was approved by the institutional review board at Claremont McKenna College and de-identified. Pole balancing at the fingertip was performed as described previously using the training protocol outlined in Milton et al. (2016). Briefly, subjects were seated in a chair while facing a blank black screen (Figure 1A). They were required to keep their back against the back of the chair. This requirement eliminates (greatly reduces) the extreme fluctuations in stick angle that precede a fall if the seated subject is allowed to move freely (Márquez et al., 2023). The pole is an oak dowel with diameter 6.35 mm, length 0.57 m (length of dowel plus 0.009 m reflective markers attached at each end). The mean balance time for Subject A was 31 s and for Subject B it was 25 s. Since the mean balance times were less than 240 s, both of these subjects are considered to be “novice” pole balancers (Milton et al., 2016). The mass of the pole plus reflective markers was 13.8 g. A high-speed motion capture system (3 Qualisys Oqus 300 cameras, 250 Hz) was used to measure the position of reflective markers during pole balancing as a function of time. Balance trials in which the fall occurs in the sagittal plane were selected for this study [70 − 80% of stick falls occur in this direction (Milton et al., 2016; Insperger and Milton, 2021)]. Data was down-sampled to 100 Hz for all our fall forecasting experiments to mimic real-world motion sensor devices, such as smartwatches and phones which typically collect data at 30–100 Hz.

### 2.2 Mathematical model for stick balancing

Stick balancing on the fingertip was modeled using an inverted pendulum-cart mechanical model along with human control force in the form of time-delayed predictor feedback (Figure 1B). The equation of motion for the stick itself is

$$\left(\begin{array}{cc} \frac{1}{3}ml^{2} & \frac{1}{2}ml\cos\theta \\ \frac{1}{2}ml\cos\theta & m+m_{0} \end{array}\right)\left(\begin{array}{c} \ddot{\theta }\\ \ddot{x} \end{array}\right)+\left(\begin{array}{c} -\frac{1}{2}mgl\sin\theta \\ -\frac{1}{2}ml{\dot{\theta }}^{2}\sin\theta \end{array}\right)=\left(\begin{array}{c} 0\\ f\left(t\right) \end{array}\right)$$ (1)

where *m* is the mass of the stick, *m*_0_ is the mass of the cart, which is equivalent to the inertia of the human arm segments plus the hand (Nagy et al., 2020), *θ* is the vertical displacement angle of the stick, $\ddot{x}$ is the acceleration of the fingertip (cart), $\ddot{\theta }$ is the angular acceleration of the stick, and *f*(*t*) is the control force. The linearized equation of motion for the control of the pendulum-cart model is

$$\left(\begin{array}{cc} \frac{1}{3}ml^{2} & \frac{1}{2}ml\\ \frac{1}{2}ml & m+m_{0} \end{array}\right)\left(\begin{array}{c} \ddot{\theta }\\ \ddot{x} \end{array}\right)+\left(\begin{array}{cc} -\frac{1}{2}mgl & 0\\ 0 & 0 \end{array}\right)\left(\begin{array}{c} \theta \\ x \end{array}\right)=\left(\begin{array}{c} 0\\ f\left(t\right) \end{array}\right).$$ (2)

Assuming predictor feedback, the control action, *f*(*t*), takes the form

$$\begin{array}{ll} f\left(t\right) & =k_{\text{p},\theta }\theta \left(t-\tau \right)+k_{\text{p},x}x\left(t-\tau \right)+k_{\text{d},\theta }\dot{\theta }\left(t-\tau \right)+k_{\text{d},x}\dot{x}\left(t-\tau \right)\\ & \;+\int_{t-\tau }^{t}k_{\text{f}}\left(t-s\right)f_{\text{PF}}\left(s\right)ds, \end{array}$$ (3)

where *k*_p,*θ*_, *k*_p,*x*_, *k*_d,*θ*_ and *k*_d,*x*_ are, respectively, the proportional and derivative control gains for *θ* and *x*, and *k*_f_ is a function related to the predictor feedback [for further details, see Insperger and Milton (2021)]. The first four terms in Eq. 3 represent the delayed state feedback, while the last term is associated with the weighted integral of the issued control force over the interval [*t* − *τ*, *t*] (Krstic, 2009). The effect of a sensory dead zone in the sagittal plane, related to difficulties with depth perception (Milton et al., 2016), was modeled as a switching component to the control. In other words, the feedback is turned on or off depending on whether *θ* is larger or smaller than a sensory threshold Π, i.e.,

$$\theta_{\text{perceived}}\left(t-\tau \right)=\left\{\begin{array}{cc} 0\; & if\;|\theta \left(t-\tau \right)|<\Pi \\ \theta \left(t-\tau \right)\; & if\;|\theta \left(t-\tau \right)|\geq \Pi . \end{array}\right.$$ (4)

However, information related to $\dot{\theta }$ and $\ddot{\theta }$ remains available (Thiel et al., 2007). The presence of this dead zone accounts for the observation that 70%–80% of human stick falls occur in the anterior-posterior direction.

The model’s equations were integrated using semi-discretization (Insperger and Stépán, 2011) because the integral term in Eq. 3 cannot be implemented analytically. In the computer code, we discretized the control force and approximated the integral by a discrete sum of delayed values with a sampling time of 10 ms. This approach is equivalent to the assumption that the nervous system behaves like a digital controller with a discrete input and a discrete output system. The solutions of this model exhibit micro-chaos (Milton et al., 2016; Milton et al., 2018; Insperger and Milton, 2021). Micro-chaotic dynamics arise generically in dynamical systems in which there is time-delayed feedback, a sensory dead zone, and frequency-dependent encoding of force. Consequently the dynamics exhibit a sensitive dependence of initial conditions and issues related to numerical algorithms and hardware. However, the stabilometric properties of the solutions, such as the feedback gains and time delays, are not affected.

#### 2.2.1 Synthetic stick falls

We consider two ways that stick falls can occur: 1) *θ* becomes too large and 2) *x* leaves the zone of possible movement, which we will discuss a little later. For stick balancing, the changes in *x* and *θ* are strongly correlated especially on time scales of 4*τ* − 5*τ*. On these time scales when a change in *x* predicts a fall then so do changes in *θ*. The largest *θ* for which balance can be maintained depends on the skill of the stick balancer: we estimated 10° for subject A and 20° for subject B.

There are two constraints placed on *x*: 1) *x* cannot be larger than the effective length, *L*, of the subject’s arm, and 2) the fingertip cannot hit the subject’s chest. The effective arm length, *L*, is shorter than the length of the arm measured from the tip of the shoulder to the tip of the out-stretched index finger. This is a result of the “slider crank” movements of the arm which are limited by the bulk of the upper arm. These constraints were modeled by assuming that the reference position *x* = 0 of the fingertip as *L*/2. Changes in *L* due to the subject leaning forward were minimized by having the subject keep their back against the back of the chair at all times.

The arm length limitations become effective at time instant *t* = *t* ^*^ if either.

• *x*(*t* ^*^) = *L*/2 and $\dot{x}\left(t^{*}\;\right)>0$ (the arm cannot stretch further) or
• *x*(*t* ^*^) = −*L*/2 and $\dot{x}\left(t^{*}\;\right)<0$ (the fingertip hits the chest).

In both cases, the fingertip position is constrained to either *x*(*t*) = *L*/2 or *x*(*t*) = −*L*/2 with $\dot{x}\left(t\right)=0$ for *t* > *t* ^*^. This corresponds to a sudden fixation in rigid body dynamics: the pole’s angular velocity changes suddenly, and the control force can be neglected compared to the arising constraint force. The pole’s angular velocity after the sudden fixation (at time *t* ^*+^) can be calculated given that the angular momentum about the fixation point (pole’s bottom) is preserved. The angular velocity just after the sudden fixation is

$$\dot{\theta }\left(t^{*+}\right)=\dot{\theta }\left(t^{*}\;\right)+\frac{2}{l}\dot{x}\left(t^{*},\;\right).$$ (5)

The pole’s angular position after the sudden fixation can be calculated by the open-loop dynamics of a pinned pendulum. Time domain simulations show that arm length limitation ends up with the pole falling within 1–2 s.

#### 2.2.2 Parameter estimation

Before generating synthetic data using the model described by Eqs 1–5, the following model parameters had to be identified: the time delay (*τ*), the sensory threshold (Π) and the four feedback control gains (*k*_p,*θ*_, *k*_p,*x*_, *k*_d,*θ*_, *k*_d,*x*_). The microchaotic nature of the data makes it difficult to estimate model parameters by directly comparing the model generated and observed time series. To overcome this problem, we used a stabilometric approach to estimate the parameters (Nagy et al., 2023). Briefly, this approach assumes that the choices of the parameters are acceptable if the model using these parameter values reproduces certain statistical properties of the data including the standard deviation of the stick angle and certain frequency dependent properties. The time delay (0.23 s) determined in this way is in good agreement with that determined by measuring the response to a mechanical perturbation (Mehta and Schaal, 2002) and to a visual blank out (Milton et al., 2016). The threshold for the sensory dead zone agrees with that estimated from time series analysis (Milton et al., 2016).

**FIGURE 2:**
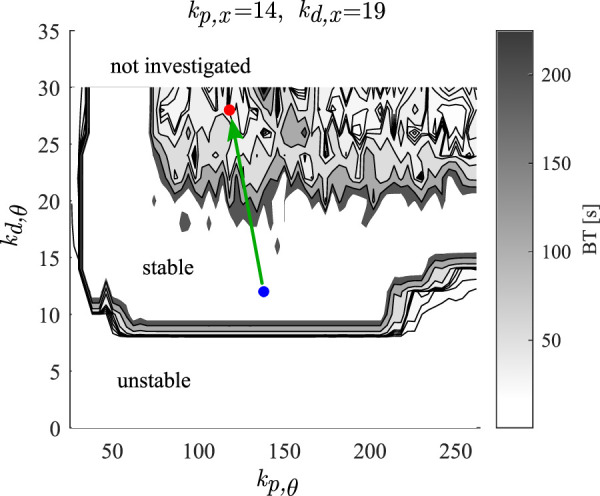
Tuning of the control gains for the simulations of the simulated data. Greyscale show the balance times, the blue dot indicates the identified control gains for balance time >240 s, and red dot indicates the tuned control gains for balance time ≈31 s.

The gains determined by the stabilometric technique are those for stable pole balancing, i.e., the balance times are greater than 240 s (see blue dot in Figure 2). We assumed that transient pole balancing resulted from alterations in the control gains for the pole angle, *k*_p,*θ*_ and *k*_d,*θ*_. The control gains for the movements for the position of the cart were not changed. Figure 2 summarizes the balance time predicted by the model as a function of *k*_p,*θ*_ and *k*_d,*θ*_ for fixed *k*_p,*x*_ and *k*_d,*x*_. Three types of behaviors can be observed: 1) unstable balancing with balance time ≤1 s, 2) stable balancing with BT ≥240 s, and 3) transient balancing with 1 s < *BT* < 240 s. The blue dot shows the values of the gains for the control of the pole angle determined using stabilometry.

We used the measured mean pole balancing time to choose the gains for the control of the pole angle generated by the model (simulated data). We estimated the mean pole balancing time by simulating data with initial angles ranging from −3° to 3° in increments of 0.001°.

**FIGURE 3:**
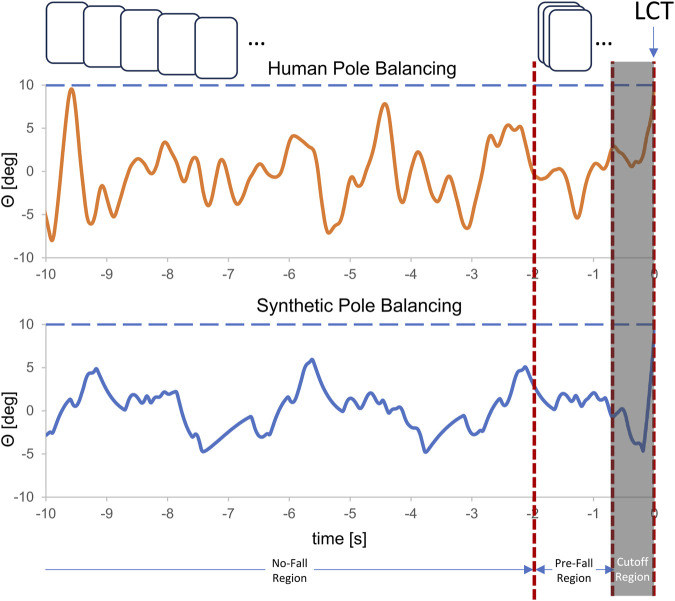
Comparison of the changes for the last 10 s of balancing in vertical displacement angle, *θ*, for synthetic data and real pole balancing data for Subject A. The data pre-processing process excluded the data points in the last portion of the signal (indicated by LCT and labeled as Cutoff region) that correspond to the start of a fall. We divide the remaining data into no fall and pre-fall which we label as 0 and 1. The generated data was further partitioned into windows, which are visually represented by boxes colored in shades of gray (indicates no-fall region) and brown (indicates pre-fall region). The horizontal dashed line indicates the *θ* when the control was lost.

The values of the gains for the simulated data were chosen to give a similar mean balance time as observed experimentally for each subject (see Table 1; Figure 3). For Subject A’s parameters, the mean balance time for simulated data was 30.4 ± 24.8 s (mean ± one standard deviation) and the measured mean balance time for 95 balancing trials was 31 ± 19.8 s. The red dot in Figure 2 shows the values of the gains. For Subject B’s the mean balance time for simulated data was 24.6 ± 9.8 s and the measured mean balance time for 21 balancing trials was 24.8 ± 13.05 s.

**TABLE 1:** Control gains estimated from real pole balancing data. Note that *N* is Newtons, *rad* is radians, *m* is meters and *s* is seconds. Π refers to the dead zone threshold.

Subject	*k*_p,*θ*_(*N*/*rad*)	*k*_d,*θ*_(*Ns*/*rad*)	*k*_p,*x*_(*N*/*m*)	*k*_d,*x*_(*Ns*/*m*)	Π (rad)
A	120	28	14	19	1.75
B	240	30	13	30	3.00

### 2.3 Data generation and pre-processing for machine learning

We configured the mathematical model using the control gains shown in Table 1. An initial angle was randomly selected from a range between [−3, 3]. The data generation process involves running six simulations, and computing the feature set: fingertip position *x*, and vertical displacement angle *θ* at each time step. A total of 1,000 balancing trials are generated in each simulation. The time point at which balance control was lost (LCT) was determined as follows: First, the largest *θ* for which the stick remained balanced was determined from all of the balance trials. Balance control was lost when the angle in the next time step (0.01 s) was larger. The data points for times greater than LCT were discarded. This is indicated to the right of the vertical orange line in Figure 3. Since our focus is on forecasting, not fall detection, we further removed the 0.75 s prior to LCT to make sure that the changes in angular displacement *θ* and fingertip position *x* were not those related to falling (i.e., truly pre-fall data). This time can also be thought of as time (buffer zone) available to activate strategies to either prevent or minimize the consequences of a fall.

The remaining data is then partitioned into no-fall and pre-fall regions and further segmented into fixed-size windows creating training instances/windows using the sliding window protocol as shown in Figure 3. In all of our experiments, each window is set to 1.28 s long which is sufficient to account for the dynamics of a pre-fall pattern. The amount of data labeled as pre-fall is determined by the time-to-predict parameter and the size of the window we used. We vary the time-to-predict parameters from 0.1 s to 1.5 s to assess how far ahead we can forecast impending fall in an unstable dynamical system.

#### 2.3.1 Balancing training data

Given that each simulation will generate a lot more data for the no-fall region than the pre-fall region, our data set is massively unbalanced. In machine learning, training with an unbalanced dataset will end up with a model that cannot be generalized. To balance our data set, we dynamically computed the stride length (i.e., the distance the window moves), within the no-fall region to match the number of windows within the pre-fall region during the sliding window generation process. The key is to first maintain a fixed stride size of 1 (or other suitable sizes) within the pre-fall region. Assuming that the length of the pre-fall region is represented by *n*, we will obtain a total of (*n* − *w* + 1) sliding windows within the pre-fall region, with a stride of 1 and a window size of *w*. Afterward, we dynamically calculate the stride size (*S*1) within the no-fall region to correspond to the number of windows in the pre-fall region using this computation:

$$S1=\frac{\left(\text{number of sliding windows in no fall region}\right)}{\left(\text{number of sliding windows in pre}-\text{fall region}\right)}$$

where, the number of sliding windows in the no-fall region is (*N* − *w* + 1). Here *N* refers to the length of the no-fall region. By adjusting the stride size within the no-fall region, the model observes a nearly equal number of data windows from both the no-fall and pre-fall regions.

The traditional or naive way of balancing the dataset is by running additional simulations, labeling the simulated data as no-fall and pre-fall after cropping off the specified number of data points in the LCT region at the end, and only saving the pre-fall section of the data for training. This is a much more computation-intensive way of data balancing and might generate inconsistent training datasets since additional pre-fall data do not have a corresponding no-fall data region. Given the temporal relationships that could arise between the corresponding no-fall and pre-fall regions, only adding in one of those regions could negatively affect the training process.

### 2.4 LSTM networks and model training

The ideal model for fall forecasting should be able to learn a nonlinear function that could accurately recognize patterns preceding falls, within a specified amount of time ahead (i.e., time to predict). We want to investigate whether a deep-learning model can be used for learning this nonlinear function, specifically the LSTM model, to predict the stick fall in human stick-balancing problems. Starting with LSTM, the simplest model for capturing temporal dynamics, we lay the groundwork for future exploration into more complex models.

**FIGURE 4:**
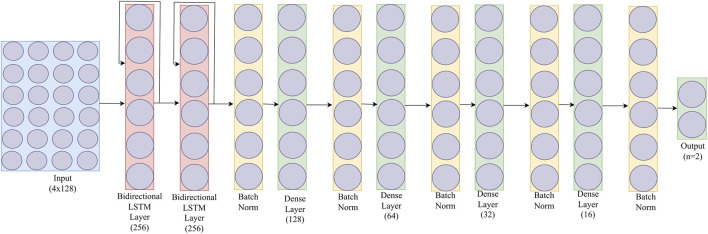
A bidirectional recurrent unit (LSTM) for fall forecasting.

Our choice of Bi-LSTM was based on its ability to learn patterns from both directions, making it contextually more aware. It also addresses the limitations of traditional LSTMs, such as the vanishing gradient problem, and enables the handling of longer temporal dependencies. Our choice is supported by other researchers. For example, in Wang et al. (2022), many variants of stacked LSTM models were used for crash prediction in a disorienting spaceflight analog balancing task from the time series data. Figure 4 shows the stacked bi-directional LSTM architecture we used with batch normalization. Incorporating a batch normalization layer stabilizes the training process, facilitating faster convergence, and enhancing model performance. Moreover, it also regularizes the model, minimizing overfitting and enhancing its ability to generalize to new data to make the model more robust.

We selected SGD (Stochastic Gradient Descent) as our optimizer since it can lead to faster convergence, which is vital for quickly learning from historical data in time series forecasting. Since our problem can be framed as a pre-fall-vs-no-fall classification, a Binary-Cross-Entropy (BCE) is chosen for the loss function. We set a large epoch size of 100 but adopt an early stopping strategy. All our models were built and trained using Keras and TensorFlow on Dell Precision 7820 Tower with 256 GB RAM, and four GeForce GTX 1080 GPUs. The hyperparameters used in our bi-directional LSTMs are presented in Table 2.

**TABLE 2:** LSTM model configuration.

Names	BiLSTM
Learning rate	0.001
Epochs	100
Batch size	128
Optimizer	SGD
Loss function	BCE

We adopt a methodical approach for fine-tuning the hyper-parameters of the model. The window size of 128 units, was chosen based on the delay inherent in our physical system. Other parameters, including the number of layers and neurons, were determined through a progressive grid search, aimed at validating our hypothesis. This search was halted upon achieving successful preliminary results, indicating the model’s potential. The learning rate, initially set through trial and error, was dynamically adjusted via a scheduler to decrease over time, optimizing the model’s performance.

These hyper-parameter adjustments had significant impacts. For instance, excessive layers or neurons increased model complexity, leading to overfitting and poor generalization. Conversely, too few layers or neurons result in less expressive model because of low parametrization, causing under-fitting. A fixed learning rate was found inadequate, as it hindered the model’s convergence to an optimal solution over time. Implementing a learning rate scheduler effectively addressed this, ensuring optimal model performance.

#### 2.4.1 Input features

The inputs to our bi-directional LSTM are the angular displacement and the position of the pole both at time *t* and at *t* − *τ*, where *τ* is the time delay in the mathematical model as discussed earlier. The vertical angular displacement is defined as the angle made by the pole with the sagittal plane. Similarly, the position of the pole at time *t* is defined by the actual position of the fingertip (on the x-axis). Data is fed one window at a time with the above four features. It is important to note that in the experiments, the dimension of the input features is defined as, *X*_*input*_ = (*θ*(*t* − *τ*), *θ*(*t*), *x*(*t* − *τ*), *x*(*t*)) such that $X_{\mathrm{input}}\in R^{d\times 4}$ , where *d* is the size of the window of data (1.28 s) for the optimal learning of pre-fall signals.

#### 2.4.2 Model validation

To determine how well our models perform and which model will perform best on unseen data in each experiment, we split the dataset into training, validation, and testing sets. In the first experiment, we trained our Bi-LSTM solely with all the limited real data we had, dividing the dataset into approximate 80/10/10 ratios for the training, validation, and test sets. For the second experiment, we train the BI-LSTM using only the synthetic data generated from the mathematical stick balancing model, we split the synthetics dataset into 90 percent for training and 10 percent for validation. Since the dataset is large, reserving 10 percent for validation is sufficient. We used the entire real dataset to test the model’s generalizability. For the third experiment, which is transfer learning, we started with a pre-trained model from the synthetic data. We split all the available real human data into training and test sets in the approximate ratio of 85/15. This division was performed based on the available number of trials for each subject. Using subject A’s as an example, this translates to 22 trials of the train and 4 trials for the test where the total available real data for subject A is 26.

In general, the data should be split in such a way that none of the data in the validation or test set appears in the training set. Data used for our experiments are balanced in terms of no-fall and pre-fall data windows. Since time to predict will affect the amount of data available for training, down-sampling of data is performed to ensure that all our experiments are using the same number of windows of no-fall and pre-fall data as used in the experiment using the smallest time-to-predict value.

#### 2.4.3 Evaluation metric

We adopted the standard metric to evaluate the performance of a machine-learning model. There are the F1 score, Precision, Recall, and Accuracy. These metrics are defined as:

$$Precision=\frac{TP}{TP+FP}$$

$$Recall=\frac{TP}{TP+FN}$$

$$F1\text{\_}Score=\frac{2\cdot Recall\cdot Precision}{Recall+Precision}$$

$$Accuracy=\frac{TP+TN}{TP+TN+FP+FN}$$

True Positive (TP) occurs when the model correctly predicts a positive instance, such as accurately forecasting a fall. True Negative (TN) is when the model correctly predicts a negative instance (non-fall for our case). False Positive is the case when the model erroneously predicts a negative data sample as positive and False Negative is the opposite case. Precision assesses the accuracy of positive prediction, and Recall, which is the same as sensitivity, quantifies the correct identification of real positives. The F1 score is a harmonic mean of precision and recall to provide a balanced measure of model performance.

In general, higher score of precision, recall, F1-score and accuracy indicate better generalization of the model. A high recall means that we do not miss predicting an impending fall (all true events are detected), while high precision indicates that we do not predict non-falls as impending falls (resulting in very low false alarms). Accuracy measures how well the model correctly forecasts impending fall and non-fall events. In a stick balance experiment or simulation, the occurrence of a fall is a critical event. Surrounding this event, the characteristics that precede the fall of the stick are significantly informative for the prediction task. We do not want to overlook any potential falls, so relying solely on accuracy as a performance metric is inadequate. The additional evaluative criteria, such as precision, recall, and F-score are all needed to comprehensively assess predictive performance.

To ensure the trained model generalizes well, the evaluation process of the model involves splitting the training data into training, validation, and test sets. The validation and test sets contain data independent from the original training data but with a similar statistical distribution. The validation set is used to assess the generalization of the model during training, and only a model that generalizes well with the validation set is selected as the final model to be tested with the test set. If a model fails to generalize during validation, hyperparameters of the model need to be tuned. For example, we added batch normalization and introduced a new technique to balance the data.

#### 2.4.4 Transfer learning

**FIGURE 5:**
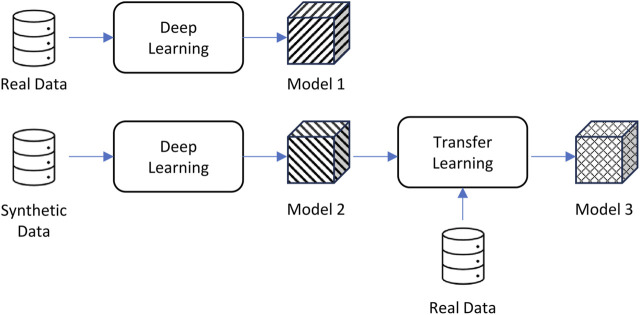
The three models used for our experiments: real data only, synthetic data only, and a combined approach.

In the computer vision world, transfer learning has been applied successfully to transfer knowledge from a model trained in an existing domain to data captured from a new domain that has a slightly different prediction target. For example, using transfer learning, you can take an existing algorithm trained to identify 50 different flowers in images and use it to create a new algorithm that can identify those 50 flowers plus 10 new types of flowers, all while reducing the training time of the new algorithm (Pan and Yang, 2010; Zhuang et al., 2021). Essentially, transfer learning uses a pre-trained model as a base for a new model of a target in a similar domain. Transfer learning allows us to refine a base model with a small amount of real human pole balancing data set and then evaluate the model on the rest of the real data. Figure 5 is a schematic diagram that illustrates this process.

We implemented the transfer learning by first initializing the network with the pre-trained weights of a saved model. We split our real pole balancing data where one set was used for the refinement of the saved model and another set was used for testing the model after refinement.

### 2.5 Experiments

#### 2.5.1 LSTM training with only real data

At first, we trained our LSTM model using only measured data captured by the motion camera from the human subject. This is to create a baseline model for comparison with other models in our experiments. We used two subjects’ data to conduct our experimental study. For subject A, there were a total of 28 data samples available. We used 26 data samples/balance trials for training and 2 samples for testing. For subject B, only 14 samples were available. We thus used 12 data samples for training and 2 for testing. We only trained one forecasting model using the smallest time to predict value (0.1 s) for each subject.

#### 2.5.2 LSTM training with only synthetic data

The goal of this experiment is to train a series of models purely using synthetic data with different time-to-predict values to assess the limit of the performance of the LSTM machine-learning algorithm for predicting impending stick falls. A MATLAB program that implemented the mathematical model described in Section 2.2 was used to generate synthetic data for subjects A and B using the estimated parameter shown in Table 1. We labeled the generated data set into no-fall and pre-fall regions, segmented them into windows of 1.28 s long, and balanced the no-fall and pre-fall windows as described earlier in Section 2.3.1. In each of the LSTM training sessions, the training set contained 1,000 samples, while the validation set contained 50 samples. This model is then tested on the small amount of real pole balancing available to us to measure how the model trained with synthetic data performs against real data. For each subject, we repeated this LSTM training six times with different batches of synthetic training data to eliminate the probability of the randomness effect. In addition, each of these LSTM training is run 15 times using a different time-to-predict value from 0.1 s to 1.5 s with a step size of 0.1.

To ensure that our results are not due to insufficient training data, we trained one LSTM model using 10,000 samples/balance trials of synthetic data. Training this large data set took 8 h on a computer with four GPUs. The model trained using this large data set performed similarly to the one trained with only 1,000 balance trials. For computational efficiency, we ran all of our remaining experiments with only 1,000 balance trials.

#### 2.5.3 Refining LSTM model via transfer learning

In this third experiment, we studied the impact of transfer learning on improving a model trained using purely synthetic data by utilizing a small amount of real human pole-balancing data. To achieve that, we refined all the models trained with synthetic data using both subject A and B’s real-human pole balancing data. As in other experiments, we split the available human balancing data into training and validation sets. The model was re-trained with a very small learning rate so that we do not drastically change the trained weights.

## 3 Results

### 3.1 Training with only real data

Table 3 presents the result of training solely on real human pole balancing data, which reveals bad performance for both subjects A and B. This model was trained using only a time-to-predict of 0.1 s, which presumably should be the best that can be done as the data is closest to the fall. These results were expected as the limited availability of data was not able to train the LSTM model effectively.

**TABLE 3:** Result of training with only a small amount of real data. Time-to-predict is 0.1 s.

	Subject A	Subject B
Precision	0.48	0.38
Recall	0.37	0.40
Accuracy	0.55	0.54

### 3.2 Training with only synthetic data

**FIGURE 6:**
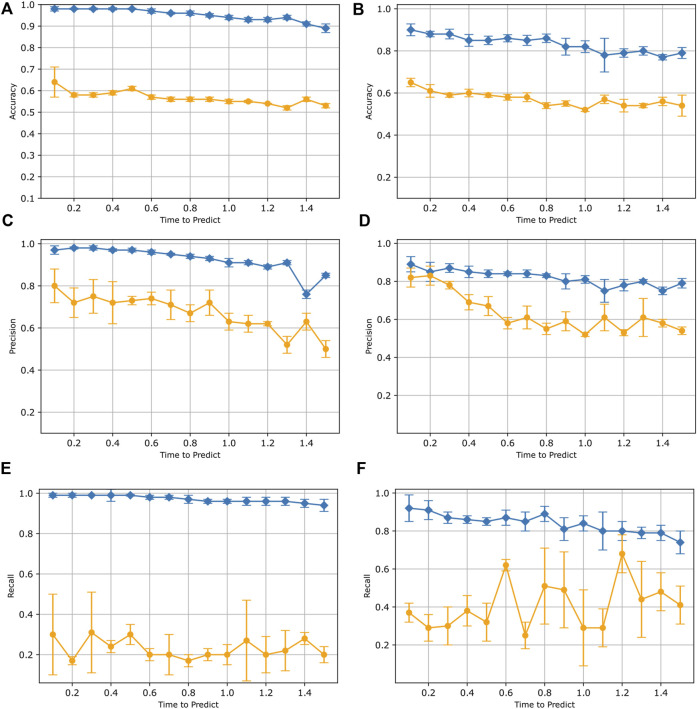
Results for training with only synthetic data. In each plot, test results on simulated data (blue) are compared to results when tested with real human data (orange). Both data from subject A **(A,C,E)** and subject B **(B,D,F)** are displayed. Each data point represents the mean accuracy **(A,D)**, precision **(B,E)**, and recall **(C,F)** values evaluated across six separate trained models. The bars indicate the standard deviation.

In Figure 6, we present the average and standard deviation of the precision, recall, and accuracy values obtained from six independently trained LSTM models for both subject A’s and B’s synthetic data. The LSTM models performed well when tested using synthetic data, but did not perform as well when tested against real human data. For synthetic data, we observe that as the time-to-predict values increase, there is a decrease in accuracy, precision, and recall scores. This trend holds true for accuracy and precision scores when dealing with real data as well. This observation applies to both Subject A’s and Subject B’s data. These results indicate that our LSTM model can perform better with a shorter time to predict value, which is expected given that the larger time-to-predict values incorporate data further away from the fall itself. The recall score for both Subject A and B’s real data appears to exhibit some variability with respect to the time to predict values. One possible explanation for this low recall could be that the synthetic data may not capture the full range of diversity and complexity found in real pole-balancing scenarios. Additionally, the model trained on synthetic data may not exhibit effective generalization when applied to real pole-balancing data.

### 3.3 Refining LSTM model through transfer learning

**FIGURE 7:**
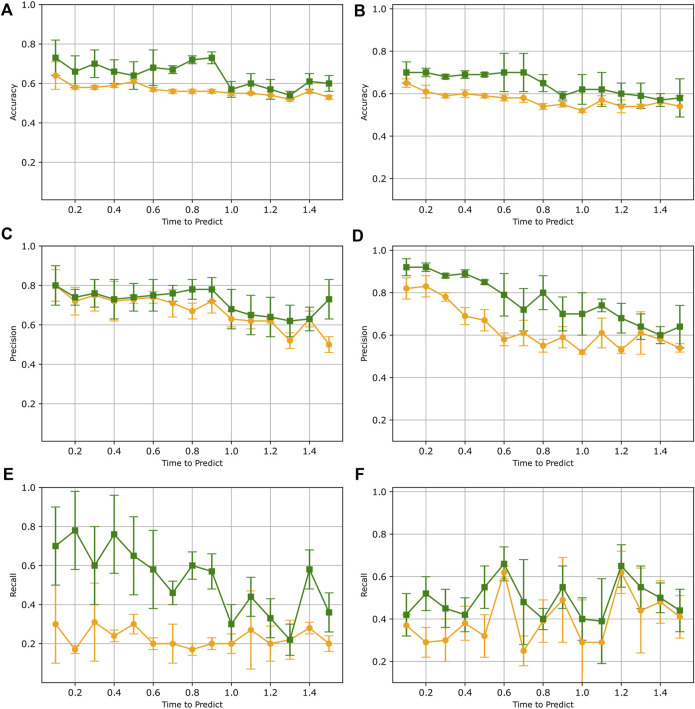
Results after refining LSTM model via transfer learning. In each plot, test results before transfer learning (orange) are compared to results when tested after transfer learning (green). Both data from subject A **(A,C,E)** and subject B **(B,D,F)** are displayed. Each data point represents the mean accuracy **(A,B)**, precision **(C,D)**, and recall **(E,F)** values evaluated across six separate trained models. The bars indicate the standard deviation.

Figure 7 summarizes the results of the LSTM model trained initially on synthetic data and refined with real human pole balancing data. As we can see from this figure, the results for both subjects A and B suggest an improvement with transfer learning. We see that for both subjects, precision, recall, and accuracy have all improved substantially from when the LSTM model was trained only on synthetic data. After training with transfer learning, the overall accuracy for both Subject A and B exhibited an improvement of approximately 10%–18%. The results also demonstrate an increase in both precision and recall for both subjects. These improvements indicate that the LSTM model, which was originally trained solely on synthetic data, now demonstrates improved generalization to real pole balancing data.

This improved generalization can be explained by the fact that transfer learning starts with a pre-trained model that already learned a sub-optimal model of the physics of stick falls. This pre-trained model greatly facilitates the learning of the dynamics of real human stick falls because the initial model is initialized with weights derived from observing a large number of examples of synthetic stick fall data. Without transfer learning, the initial model is randomly initialized, and coupled with a limited amount of data, the training cannot achieve a model with high accuracy.

When evaluating accuracy and precision scores on data from both subjects, the results after training with transfer learning also reveal a subtle pattern where the scores gradually decrease as the time to predict values increases. This phenomenon is expected as an increasing time-to-predict means that the window being examined is farther away from the fall. As with any forecasting problem, there is more uncertainty when predicting far into the future compared to predicting closer to the present. This is even more true with complex, chaotic dynamical systems as the smallest fluctuations can have rapid divergence in a few time steps. We recognize that our model will not be useful for predicting stick fall with a long anticipation time. However, the short anticipation of 2.35 s that we achieved is sufficient for alerting users or triggering preventive measures to stabilize it. The particular machine learning architecture chosen here is relatively simplistic, and the data is limited. Future studies may show that improving the LSTM architecture may yield longer anticipation times.

We notice that while our accuracy and precision are relatively high, our recall is low when testing against the model re-trained with a small amount of human balancing data. In our binary classification of no-fall or pre-fall, a threshold of 0.5 is being used. Recall can be improved by fine-tuning the hyper-parameters of the LSTM model such as the threshold value, the window size, and the systematic investigation of the trade-off between precision and recall in future work.

## 4 Discussion

While traditional machine learning techniques have been used by many researchers for the forecasting of adverse events (Zgonnikova et al., 2016; Varshneya et al., 2021; Wang et al., 2022; Márquez et al., 2023), recent studies have shown that when data sets are complex and dynamic, deep machine learning techniques always surpass these approaches (Martí-Juan et al., 2020). In our previous study, we compared traditional machine learning techniques (Support Vector Machine, Random Forest, Naive Bayes) against deep learning architectures for fall detection and found better performance from deep learning approaches Mauldin et al. (2018). In this paper, we investigated in depth the use of transfer learning and a stacked LSTM deep learning model to train the forecasting model for stick fall.

We addressed the data scarcity problem in trying to forecast impending falls using deep learning by showing that data generated by a mathematical model for stick balancing can be used to effectively train a deep machine learning algorithm to predict stick falls. We balanced the massively unbalanced data using an innovative balancing technique. In particular, our results show that by combining mathematically generated data with a limited amount of real data using transfer learning, the ability to forecast falls is increased significantly over that obtained with training using a small amount of available real data alone. Our approach resulted in a fall forecasting model that is 60%–70% reliable up to 2.35 s. This increase in forecasting times increases the time available for activating strategies to minimize the effects of a fall (Nemeth et al., 2022; Márquez et al., 2023).

As stated earlier, we noticed that as the time-to-predict increases, the accuracy and prediction decrease. One way to understand this is that the latent space representations of the pre-fall and no-fall data windows become more similar to some extent, and the “signatures” indicative of the dynamics of pre-fall may not have fully manifested because the time-to-predict is too large. This provides us with an interesting direction for future research in exploring the specific dynamical changes that occur as the fall approaches. One possible future direction is thus to train our model with a contrastive supervised learning (Khosla et al., 2020) approach. By this method, we first train our model to distinguish between the pre-fall data and no-fall data by increasing the distance between the latent space representation and later refine the model by further training to classify the pre-fall and no-fall. Another direction is to compare the effectiveness of the forecasting model trained with the mathematically generated data versus other techniques such as Diffusion (Tashiro et al., 2021), GAN (Ganin et al., 2016), and statistical approaches when data scarcity is a problem. This will confirm the importance of empirical modeling to capture “key signatures” of stick fall as compared to those reproduced by the popular generative AI approaches.

The results that we have when tested against simulated data indicate that the stacked LSTM architecture is able to accurately forecast complex and chaotic dynamical systems. However, when we tested against real human data, the low recall suggests that our model can continue to be improved in its anticipation of a fall. The precision indicates that when the model suggests a fall is indeed impending, we can expect a fall to occur. Unfortunately, for about 30% of the time, the models are unable to distinguish between pre-fall and no-fall windows. These results suggest that while this approach has indeed improved our ability to anticipate fall events despite the complexity and chaotic nature of the data, discrepancies between the simulated data and real data remain.

Indeed, in our approach, the matching of the mathematical model to human data was purposely designed to take into account the individual’s differences in mean balance time, the gains for time-delayed predictive feedback, and their sensory dead zones. However, when plotting the real human data and synthetic data side by side, we can visualize the differences in their dynamical properties as shown in Figure 3. This suggests that there may be other factors important to anticipating impending stick falls that the LSTM has learned to anticipate, that are not necessarily found in the real data.

The above points are critically important since many attempts to generate synthetic data could potentially fail for the same reason. For example, although synthetic data generation by Generative Adversarial Networks (GANs) could potentially construct time-series data that have similar statistical properties as real data when aggregated, GANs may fail to accurately capture key features or signatures when examining a single trajectory that are indicative of an impending collapse or critical transition. Our observation that LSTM is able to forecast adverse events in a micro-chaotic dynamical system suggests that such approaches rely more on the probabilistic properties of such systems is as yet an unidentified manner. Without having some insights into the appropriate metrics that describe these key features, synthetic data generators may instead construct data that are very accurate in every feature except the signatures themselves. The fact that human pole balancers are able to sense when they are about to lose control suggests that a signature does exist, and thus there are features that should be matched between synthetic data and real human data. Future directions may explore the use of alternative metrics to match real data with those generated through mathematical models including decomposition-based, statistical generative models and alternative embedding spaces (Wen et al., 2021).

## Data Availability

The datasets generated for this study, as well as the code base for this analysis can be found at https://github.com/jtkchang/pole-balancing.

## References

[B1] Cabrera J. L. Milton J. G. (2002). On-off intermittency in a human balancing task. Phys. Rev. Lett. 89, 158702. 10.1103/PhysRevLett.89.158702 12366030

[B2] Cabrera J. L. Milton J. G. (2004a). Human stick balancing: tuning Lèvy flights to improve balance control. Chaos Interdiscip. J. Nonlinear Sci. 14, 691–698. 10.1063/1.1785453 15446980

[B3] Cabrera J. L. Milton J. G. (2004b). Stick balancing: on-off intermittency and survival times. Nonlinear Stud. 11, 305–317.

[B4] Chen H. Lundberg S. M. Erion G. Kim J. H. Lee S.-I. (2021). Forecasting adverse surgical events using self-supervised transfer learning for physiological signals. npj Digit. Med. 4, 167–213. 10.1038/s41746-021-00536-y 34880410 PMC8654960

[B5] Çiçek E. Gören S. Memik G. (2022). Physical activity forecasting with time series data using Android smartphone. Pervasive Mob. Comput. 82, 101567. 10.1016/j.pmcj.2022.101567

[B6] Ganin Y. Ustinova E. Ajakan H. Germain P. Larochelle H. Laviolette F. (2016). Domain-adversarial training of neural networks. J. Mach. Learn. Res. 17, 189–209. 10.1007/978-3-319-58347-1_10

[B7] Gilpin W. Huang Y. Forger D. B. (2020). Learning dynamics from large biological data sets: machine learning meets systems biology. Curr. Opin. Syst. Biol. 22, 1–7. 10.1016/j.coisb.2020.07.009

[B8] Insperger T. Milton J. (2014). Sensory uncertainty and stick balancing at the fingertip. Biol. Cybern. 108, 85–101. 10.1007/s00422-013-0582-2 24463637

[B9] Insperger T. Milton J. (2021). Delay and uncertainty in human balancing tasks. Lecture notes on mathematical modelling in the life sciences. New York, NY: Springer International Publishing. 10.1007/978-3-030-84582-7

[B10] Insperger T. Stépán G. (2011). Semi-discretization for time-delay systems: stability and engineering applications. Germany: Springer Science & Business Media.

[B11] Kapteyn M. G. Knezevic D. J. Willcox K. (2020). “Toward predictive digital twins via component-based reduced-order models and interpretable machine learning,” in AIAA scitech 2020 forum (Orlando, FL: American Institute of Aeronautics and Astronautics). 10.2514/6.2020-0418

[B12] Khosla P. Teterwak P. Wang C. Sarna A. Tian Y. Isola P. (2020). Supervised contrastive learning. Adv. Neural Inf. Process. Syst. (Curran Assoc. Inc) 33, 18661–18673.

[B13] Krstic M. (2009). *Delay Compensation for nonlinear, adaptive, and PDE systems*. Systems & control: foundations & applications. Boston: Birkhäuser Boston. 10.1007/978-0-8176-4877-0

[B14] Márquez M. R. Gutiérrez E. D. Álvarez J. S. M. Milton J. G. Cabrera J. L. (2023). Machine learning forecasting of extreme fluctuations in a human balancing task. Knowledge-Based Syst. 280, 111000. 10.1016/j.knosys.2023.111000

[B15] Martí-Juan G. Sanroma-Guell G. Piella G. (2020). A survey on machine and statistical learning for longitudinal analysis of neuroimaging data in Alzheimer’s disease. Comput. Methods Programs Biomed. 189, 105348. 10.1016/j.cmpb.2020.105348 31995745

[B16] Mauldin T. R. Canby M. E. Metsis V. Ngu A. H. H. Rivera C. C. (2018). SmartFall: a smartwatch-based fall detection system using deep learning. Sensors 18, 3363. 10.3390/s18103363 30304768 PMC6210545

[B17] Mehta B. Schaal S. (2002). Forward models in visuomotor control. J. Neurophysiology 88, 942–953. 10.1152/jn.2002.88.2.942 12163543

[B18] Milton J. Insperger T. (2019). Acting together, destabilizing influences can stabilize human balance. Philosophical Trans. R. Soc. A Math. Phys. Eng. Sci. 377, 20180126. 10.1098/rsta.2018.0126 PMC6661324 31329069

[B19] Milton J. Meyer R. Zhvanetsky M. Ridge S. Insperger T. (2016). Control at stability’s edge minimizes energetic costs: expert stick balancing. J. R. Soc. Interface 13, 20160212. 10.1098/rsif.2016.0212 27278361 PMC4938085

[B20] Milton J. G. Insperger T. Cook W. Harris D. M. Stepan G. (2018). Microchaos in human postural balance: sensory dead zones and sampled time-delayed feedback. Phys. Rev. E 98, 022223. 10.1103/PhysRevE.98.022223 30253531

[B21] Nagy D. J. Bencsik L. Insperger T. (2020). Experimental estimation of tactile reaction delay during stick balancing using cepstral analysis. Mech. Syst. Signal Process. 138, 106554. 10.1016/j.ymssp.2019.106554

[B22] Nagy D. J. Milton J. G. Insperger T. (2023). Controlling stick balancing on a linear track: delayed state feedback or delay-compensating predictor feedback? Biol. Cybern. 117, 113–127. 10.1007/s00422-023-00957-w 36943486 PMC10160210

[B23] Nemeth B. van der Kaaij M. Nelissen R. van Wijnen J.-K. Drost K. Blauw G. J. (2022). Prevention of hip fractures in older adults residing in long-term care facilities with a hip airbag: a retrospective pilot study. BMC Geriatr. 22, 547. 10.1186/s12877-022-03221-1 35773627 PMC9245388

[B24] Ong C. F. Geijtenbeek T. Hicks J. L. Delp S. L. (2019). Predicting gait adaptations due to ankle plantarflexor muscle weakness and contracture using physics-based musculoskeletal simulations. PLOS Comput. Biol. 15, e1006993. 10.1371/journal.pcbi.1006993 31589597 PMC6797212

[B25] Pan S. J. Yang Q. (2010). A survey on transfer learning. IEEE Trans. Knowl. Data Eng. 22, 1345–1359. 10.1109/TKDE.2009.191

[B26] Smirnov Y. Smirnov D. Popov A. Yakovenko S. (2021). Solving musculoskeletal biomechanics with machine learning. PeerJ Comput. Sci. 7, e663. 10.7717/peerj-cs.663 PMC8409332 34541309

[B27] Tashiro Y. Song J. Song Y. Ermon S. (2021). CSDI: conditional score-based diffusion models for probabilistic time series imputation. Adv. Neural Inf. Process. Syst. (Curran Assoc. Inc.) 34, 24804–24816.

[B28] Thiel A. Greschner M. Eurich C. W. Ammermüller J. Kretzberg J. (2007). Contribution of individual retinal ganglion cell responses to velocity and acceleration encoding. J. Neurophysiology 98, 2285–2296. 10.1152/jn.01342.2006 17596411

[B29] Varshneya M. Mei X. Sobie E. A. (2021). Prediction of arrhythmia susceptibility through mathematical modeling and machine learning. Proc. Natl. Acad. Sci. 118, e2104019118. 10.1073/pnas.2104019118 34493665 PMC8449417

[B30] Walker M. A. Gurev V. Rice J. J. Greenstein J. L. Winslow R. L. (2017). Estimating the probabilities of rare arrhythmic events in multiscale computational models of cardiac cells and tissue. PLOS Comput. Biol. 13, e1005783. 10.1371/journal.pcbi.1005783 29145393 PMC5689829

[B31] Wang Y. Tang J. Vimal V. P. Lackner J. R. DiZio P. Hong P. (2022). Crash prediction using deep learning in a disorienting spaceflight analog balancing task. Front. Physiology 13, 806357. 10.3389/fphys.2022.806357 PMC8832067 35153834

[B32] Wen Q. Sun L. Yang F. Song X. Gao J. Wang X. (2021). “Time series data augmentation for deep learning: a survey,” in Proceedings of the Thirtieth International Joint Conference on Artificial Intelligence, China, 19-27 August 2021 (IEEE), 4653–4660. 10.24963/ijcai.2021/631

[B33] Zgonnikova I. Zgonnikov A. Kanemoto S. (2016). “Stick must fall: using machine learning to predict human error in virtual balancing task,” in IEEE 16th International Conference on Data Mining Workshops (ICDMW), USA, 12-15 Dec. 2016 (IEEE). 173–177. 10.1109/ICDMW.2016.0032

[B34] Zhuang F. Qi Z. Duan K. Xi D. Zhu Y. Zhu H. (2021). A comprehensive survey on transfer learning. Proc. IEEE 109, 43–76. 10.1109/JPROC.2020.3004555

